# Impact of Molar Incisor Hypomineralization on Oral Health-Related Quality of Life in Teenagers and Parental Perception: A Meta-Analysis

**DOI:** 10.1155/ijod/3425899

**Published:** 2025-11-24

**Authors:** Sara Hashemi, Narjes Amrollahi

**Affiliations:** ^1^Dental Students' Research Committee, School of Dentistry, Isfahan University of Medical Sciences, Isfahan, Iran; ^2^Dental Research Center, Department of Pediatric Dentistry, Dental Research Institute, School of Dentistry, Isfahan University of Medical Sciences, Isfahan, Iran

**Keywords:** adolescent, children, molar incisor hypomineralization, parents, quality of life

## Abstract

**Background:**

The aim of this study was to assess the impact of molar incisor hypomineralization (MIH) on oral health-related quality of life (OHRQoL) in 11–14 years old children and parents/caregivers' perception.

**Materials and Methods:**

Articles published up to December 2023 were searched in Scopus, Web of Science, and PubMed databases. From 127 articles found initially, 20 studies were considered. Finally, 11 articles were eligible to be included, of which nine articles entered to meta-analysis. Six studies using the Child Perceptions Questionnaire (CPQ) 11–14 and three studies using the Parental–Caregiver's Perceptions Questionnaire (P-CPQ) contributed to meta-analyses.

**Results:**

In MIH affected children, the total score of CPQ 11–14 with the pooled mean of 13.56 (95% confidence interval [CI]: 7.64–19.48; *p*-value <0.001) and oral symptoms and functional limitation domains with the pooled means of 5.29 (CI: 2.83–7.74; *p*-value <0.001) and 3.04 (95% CI: 0.63–5.46; *p*-value = 0.001), respectively, increased significantly. However, the increase of emotional and social well-being domains with the pooled mean of 2.99 (CI: 0.02–5.97; *p*-value = 0.05) and 2.26 (CI:−0.35 to 4.86; *p*-value = 0.09), respectively, was not significant. The results revealed that in children with MIH, no significant relation was observed in the total score of P-CPQ with the pooled mean of 9.86 (CI: −0.76 to 20.48; *p*-value = 0.07) and all domains.

**Conclusions:**

MIH decreased OHRQoL in teenagers and significantly affected all domains except for emotional and social well-being. Parents/caregivers believed MIH did not affect OHRQoL.

## 1. Introduction

Molar incisor hypomineralization (MIH) is a common developmental enamel defect affecting children, characterized by unknown etiology and presenting unique clinical challenges to oral health practitioners [1, 2]. Its global prevalence stands at 12.9% (95% confidence interval [CI]: 11.7%–14.3%) [3–5]. MIH typically impacts at least one first permanent molar, and occasionally permanent incisors. Clinically, MIH manifests as enamel fragility, increased caries susceptibility, sensitivity, and compromised restorations due to poor material adhesion, secondary caries, and generally weakly mineralized enamel, which can ultimately necessitate extraction [6].

Hypomineralization results in distinct opacities of varying sizes and colors (white, yellowish, or brown), predominantly on the occlusal surface of molars and buccal surface of incisors. Enamel with more porous features is particularly prone to post-eruptive breakdown, leading to atypical cavities or complete crown destruction. Severe carious destruction or pulpal involvement may render molars unrestorable, making extraction a last resort, often necessitating subsequent orthodontic planning [7]. These problems can significantly affect individuals' lives, leading to adverse esthetic and social consequences [8].

In recent decades, the oral health of children and adolescents has improved substantially. Consequently, oral health-related quality of life (OHRQoL) has garnered increasing attention due to its profound connection with daily activities [9]. OHRQoL reflects how an individual's oral health impacts their daily functioning and overall well-being, encompassing domains such as oral symptoms, functional limitation, emotional and social well-being [10]. OHRQoL is valuable not only for dental research and the measurement of patient-centered clinical outcomes, but it also provides critical data for dental public health management [11]. Literature indicates that enamel defects negatively impact children's quality of life by altering both the esthetics and function of the affected teeth [12].

Several studies have investigated the effect of MIH on OHRQoL in teenagers. Some studies suggest a detrimental impact of MIH on OHRQoL [8, 13], while other studies report no significant association [14, 15]. In addition to children's self-reports, parents' or caregivers' perceptions serve as a complementary data source [16]. Studies assessed parents/caregivers' perception about the impact of MIH on their children's quality of life have yielded conflicting results [17, 18]. A previous study [19], in 8–10-year-old children, revealed that MIH negatively affected the scores of the Child Perceptions Questionnaire (CPQ) 8–10 questionnaire.

These discrepancies within the existing literature highlight the necessity for a comprehensive analysis of available literature to better understand these relations. This systematic review and meta-analysis were conducted to evaluate the probable effect of MIH on OHRQoL in teenagers, as well as parents/caregivers' perception about this impact.

## 2. Materials and Methods

This systematic review was prepared and conducted based on the Preferred Reporting Items for Systematic Reviews and Meta-Analysis Protocol (PRISMA) and was approved by the ethics committee of Isfahan University of Medical Sciences, Isfahan, Iran, with the following ID number: IR.MUI.DHMT.REC.1403.045.

### 2.1. Inclusion and Exclusion Criteria

This research question was formulated utilizing the Population, Exposure, and Outcome (PEO) framework. The study encompassed case–control, cross-sectional, and preintervention clinical trial data pertaining to OHRQoL. Specifically, the included studies focused on children aged 11–14 years and their parents/caregivers (Population), who were affected by MIH (exposure), to assess the subsequent impact on their OHRQoL (outcome).

This review included English-language studies investigating the impact of MIH on OHRQoL as reported by parents/caregivers or teenagers. Case reports, letters to the editor, pilot or cohort studies, any type of reviews, and articles published in languages other than English were excluded. Furthermore, studies that did not diagnose MIH, failed to assess OHRQoL using validated indicators, or exclusively reported on the perceptions of children younger than 10 years were also not included.

### 2.2. Search Protocol

A comprehensive search of three databases (Scopus, Web of Science, and PubMed) was conducted up to December 2023. The search queries integrated Medical Subject Headings (MeSH) keywords and their corresponding synonyms, along with free-text phrases, to identify relevant articles. The Key search terms included “molar incisor hypomineralization,” “quality of life,” and “oral health.” The retrieved references were then assessed against the defined eligibility criteria. Electronic database searches were executed using a combination of key search terms: (“Life Quality” or “Health Related Quality Of Life” or “Health-Related Quality Of Life” or “HRQOL”) AND (“Dental Enamel Hypoplasia” OR “Enamel Hypoplasia” OR “Hypoplastic Enamel” OR “Dental Hypoplasia”).

### 2.3. Study Selection

Initially, the duplicate articles were removed. Subsequently, the title and abstract of the remaining articles were reviewed. The eligibility of the articles was then evaluated in full text by two independent researchers (Sara Hashemi and Narjes Amrollahi). Finally, a hand-searching process was employed, involving a review of the references cited in the included articles and key journals. Studies that did not meet the inclusion criteria were excluded. The inter-rater reliability, measured by the correlation coefficient between the two authors' search outcomes, was 0.92 for the abstract screening and 0.99 for the full-text review.

Two independent reviewers extracted qualitative and quantitative data from the selected studies. The data extracted by two independent reviewers included: author's name and publication year, Study design, age, sex, and number of participants with MIH, MIH diagnosis criteria, severity of MIH, number of children with mild or severe MIH, OHRQoL measurement tool, and the study conclusion.

### 2.4. Risk of Bias Assessment

The quality of the included studies was assessed using the Newcastle–Ottawa Quality Assessment Scale (NOS) [20]. For clinical studies, the same checklist was used to determine the risk of bias, as only preintervention data were utilized. According to NOS classification, studies were categorized into three groups based on their methodological quality: good (scores 6–9), medium (scores 3–5), and low (scores ≤2).

### 2.5. Statistical Methods

Initially, the mean and standard deviation for the intended outcomes from each study were collected. The overall effect size was reported using the Mean with a 95% CI. Depending on the heterogeneity observed among the studies, as determined by the *I*^2^ index and *Q* statistic [21], either a random-effects or fixed-effects model was employed to combine the results. Egger's regression test and the “trim and fill” method were utilized to assess potential publication bias [22]. The statistical analyses were performed using Stata (StataCorp. 2021. Stata Statistical Software: Release 17. College Station, TX, USA).

## 3. Results

### 3.1. Selection of Studies

**Figure 1:**
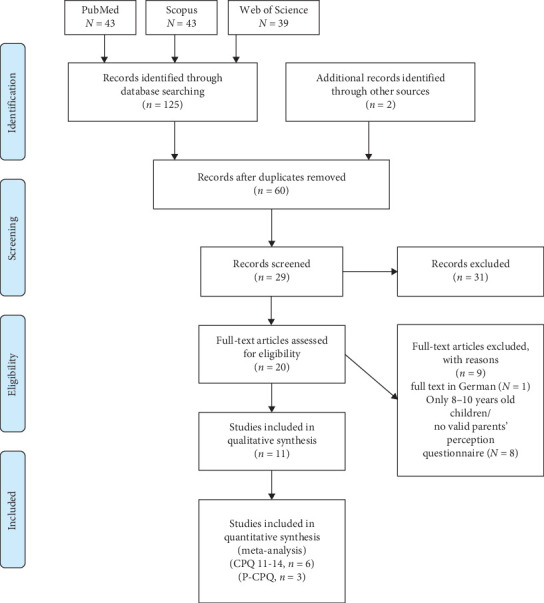
PRISMA flowchart.

Of the 127 articles retrieved initially, 67 articles were removed due to duplication. From the remaining 60 studies, 31 studies were excluded after reviewing their titles and abstracts. A further 18 articles were excluded during the full-text review. One article was excluded due to being published in German. The remaining studies were excluded because they focused on a younger age group (8–10 years) or lacked a validated questionnaire for assessing parental perceptions. Ultimately, 11 articles met the eligibility criteria, with nine of these being included in the meta-analysis (six studies for the CPQ11-14 and three studies for the Parental–Caregiver's Perceptions Questionnaire [P-CPQ]). A detailed flow diagram illustrating the search strategy and reasons for exclusion is presented in Figure 1.

### 3.2. Studies Characteristics

This review included 11 studies originating from seven different countries. The age range of participants across these studies was between 6 and 16 years. Of the 11 included studies, nine were cross-sectional [8, 13, 15, 17, 18, 23–26], one was a case–control study [14], and one was a clinical trial [27]. The specific characteristics and outcomes of the included articles are detailed in Table 1. According to the NOS, all 11 studies demonstrated high methodological quality, indicating a low risk of bias.

### 3.3. MIH Diagnosis

The MIH diagnosis tool was predominantly the European Academy of Pediatric Dentistry (EAPD) criteria from 2003 [28]. However, Freitas-Fernandez et al. [15] and Tugcu et al. [25] utilized the Ghanim grading criteria [29]. Additionally, the Developmental Defects of Enamel (DDE) index [30] was employed in studies by Arrow et al. [17, 23].

### 3.4. OHRQoL Evaluation

Among the 11 studies, seven employed the CPQ 11–14 [8, 13–15, 18, 23, 25], while three used the CPQ 8–10. The Child Oral Health Impact Profile (COHIP) and the Child Oral Impacts on Daily Performances (C-OIDP) questionnaires were used in only one study [26]. The P-CPQ was utilized in four studies; three of these also used the CPQ 11–14 questionnaire [8, 15, 18], while one study [17] exclusively used the P-CPQ. Six studies utilizing the CPQ 11–14 and three studies using the P-CPQ were included in the meta-analysis.

### 3.5. Meta-Analysis

#### 3.5.1. CPQ 11–14

Seven studies utilized CPQ 11–14 [8, 13–15, 18, 23, 25]. One of these studies [15] was excluded from the meta-analysis because its data were reported as percentages, precluding analysis using mean and standard deviation. Portella et al. [14] reported only the total, not the domain scores. Data from the preintervention phase were extracted from Tugcu et al.'s [25] study. Furthermore, one study [23] presented data from two distinct groups based on severity, and the results of these two groups were listed separately in the meta-analysis. Consequently, six studies using the CPQ 11–14 questionnaire, with a combined sample size of 950 participants, were included in the meta-analysis. Of these, five studies were used for domain-specific analyses. The domains of the CPQ 11–14 questionnaire were analyzed individually. The number of studies using other types of quality of life questionnaires was insufficient for statistical analysis.

#### 3.5.2. P-CPQ

Four studies employed the P-CPQ questionnaire to assess parental perception [8, 15, 17, 18]. In one study [15], although the P-CPQ was used, the results were reported as a regression, making them statistically incomparable to the findings of other studies. Of the remaining three studies, Dantas-Neta et al. [8] did not report the social-wellbeing and emotional-wellbeing scores separately; instead, they reported a combined score for both, which excluded this study from the meta-analysis concerning these two specific domains.

#### 3.5.3. Relationship Between MIH and the Total Score of CPQ 11–14 and Its Domains

Six studies [8, 13, 14, 18, 23, 25] with similar methodology evaluated the effect of MIH on the total score of OHRQoL by the CPQ 11–14 questionnaire, of which five of them [8, 13, 18, 23, 25] assessed its domains with similar methodology.

Heterogeneity in the analysis of total score CPQ11-14 (*I*^2^ = 0.00%, *p*-value = 0.88) and its domains, including oral symptom (*I*^2^ = 0.00%, *p*-value = 0.90), functional limitation (*I*^2^ = 0.00%, *p*-value = 0.88), emotional (*I*^2^ = 0.00%, *p*-value = 0.92), and social well-being (*I*^2^ = 0.00%, *p*-value = 0.98) was not observed.

In children affected by MIH, a statistically significant increase was noted in the total CPQ11−14 score, with a pooled mean of 13.56 (95% CI: 7.64–19.48; *p*-value <0.001). Similarly, the oral symptoms and functional limitation domains showed significant increases, with pooled means of 5.29 (CI: 2.83–7.74; *p*-value <0.001) and 3.04 (95% CI: 0.63–5.46; *p*-value = 0.001), respectively. However, the increases in the emotional and social well-being domains were not statistically significant, with pooled means of 2.99 (CI: 0.02–5.97; *p*-value = 0.05) and 2.26 (CI: −0.35 to 4.86; *p*-value = 0.09), respectively.

**Figure 2:**
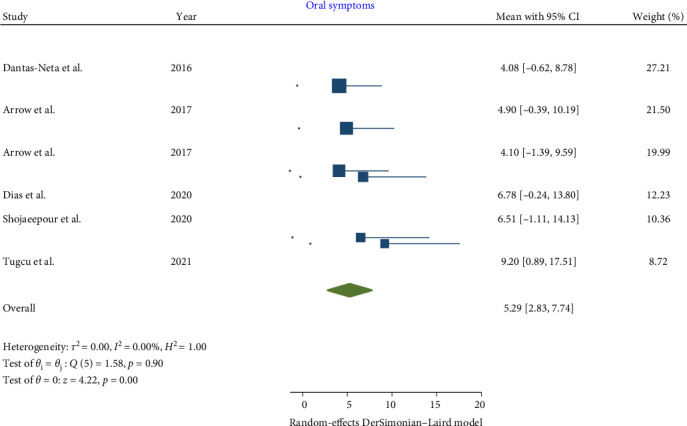
Forest plot of oral symptom domain scores of CPQ 11–14.

**Figure 3:**
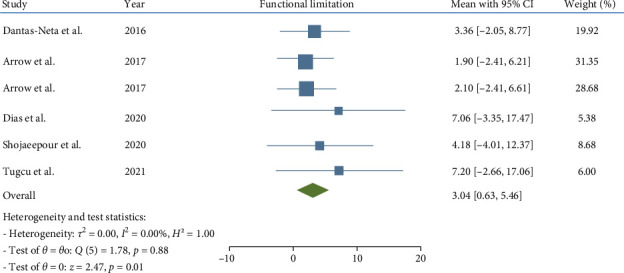
Forest plot of functional limitation domain scores of CPQ 11–14.

**Figure 4:**
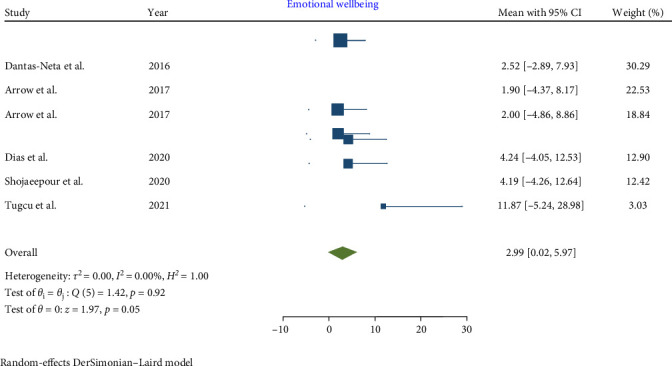
Forest plot of emotional well-being domain scores of CPQ 11–14.

**Figure 5:**
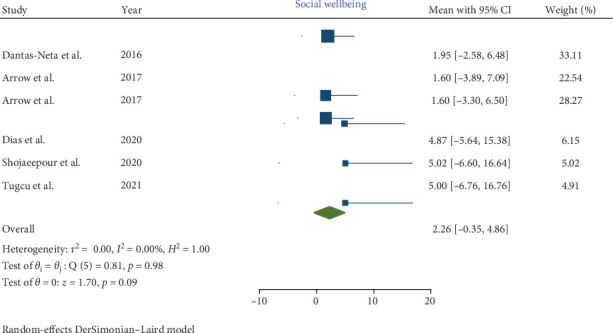
Forest plot of social well-being domain scores of CPQ 11–14.

**Figure 6:**
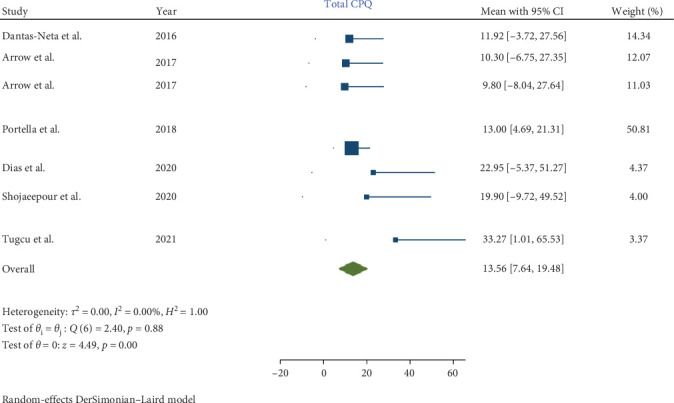
Forest plot of total scores of CPQ 11–14.

Consequently, the total CPQ11−14 score, along with the oral symptoms and functional limitation domain scores, were significantly higher in teenagers with MIH (Figures 2–6). Egger's test, used to evaluate publication bias, indicated no significant bias for the total CPQ11−14 score or its domains, including oral symptoms (*p*-value = 0.239), functional limitations (*p*-value = 0.195), emotional well-being (*p*-value = 0.265), and social well-being (*p*-value = 0.388).

#### 3.5.4. Relationship Between MIH and the Total Score of P-CPQ and Its Domains

Three studies [8, 17, 18], employing comparable methodologies, investigated the impact of MIH on the total score P-CPQ and its oral symptom and functional limitation domains. Only two of these studies [17, 18] assessed the social and emotional well-being domains.

No significant heterogeneity was detected in the meta-analysis of total score P-CPQ (*I*^2^ = 0.00%, *p*-value = 0.67) and its domains, including oral symptom (*I*^2^ = 0.00%, *p*-value = 0.78), functional limitation (*I*^2^ = 0.00%, *p*-value = 0.79), emotional (*I*^2^ = 0.00%, *p*-value = 0.45), and social well-being (*I*^2^ = 0.00%, *p*-value = 0.64). The results indicated that in children with MIH no significant increase was observed in the total score of P-CPQ with the pooled mean of 9.86 (95% CI: −0.76 to 20.48; *p*-value = 0.07) and all domains including oral symptoms, functional limitation, emotional and social well-being with the pooled means of 2.65 (95% CI: 0.55–5.86; *p*-value = 0.10), 3.09 (95% CI: −0.70 to 6.88; *p*-value = 0.11), 1.98 (95% CI: −2.59 to 6.55; *p*-value = 0.40), and 1.71 (95% CI: −2.66 to 6.08; *p*-value = 0.44), respectively.

**Figure 7:**
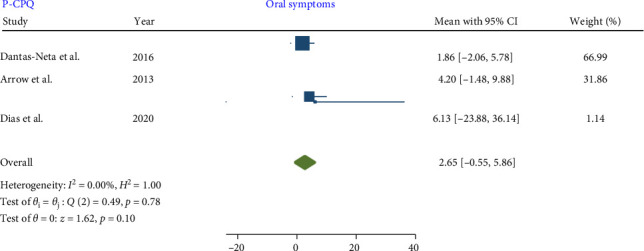
Forest plot of oral symptom domain scores of P-CPQ.

**Figure 8:**
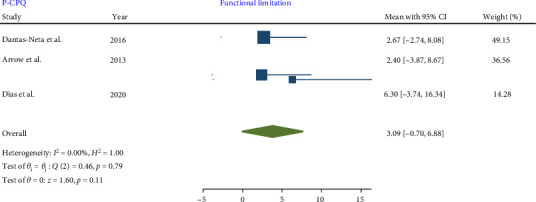
Forest plot of functional limitation domain scores of P-CPQ.

**Figure 9:**
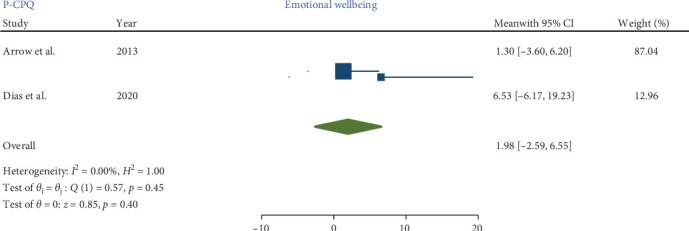
Forest plot of emotional well-being domain scores of P-CPQ.

**Figure 10:**
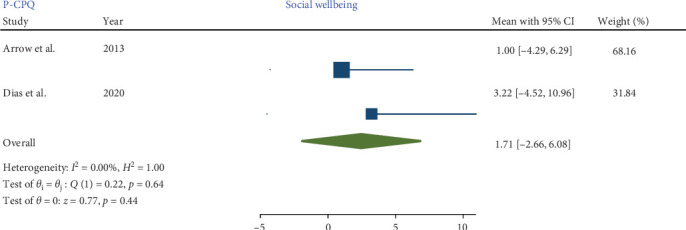
Forest plot of social well-being domain scores of P-CPQ.

**Figure 11:**
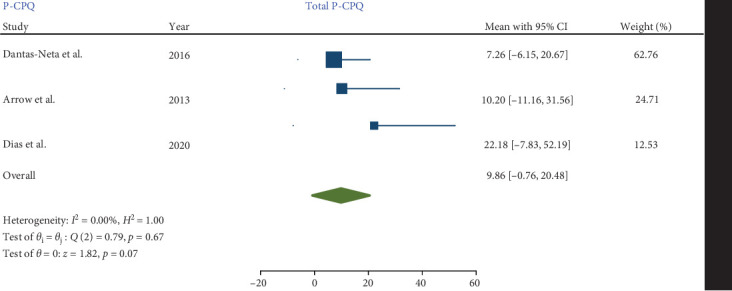
Forest plot of total scores of P-CPQ.

Therefore, based on parental/caregiver perceptions, the overall P-CPQ score and its domains were not significantly affected in children with MIH (Figures 7–11). Egger's test results for publication bias were not significant for the total CPQ11-14 score (*p*-value = 0.404) and its oral symptom (*p*-value = 0.681) and functional limitation (*p*-value = 0.523) domains.

## 4. Discussion

MIH presents as a prevalent developmental enamel defect observed in children, characterized by distinct opacities affecting first permanent molars and incisors. While its precise etiology remains elusive, affected enamel exhibits heightened susceptibility to posteruptive breakdown, potentially leading to atypical caries or, in severe instances, complete coronal destruction. Furthermore, teeth affected by MIH frequently display significant sensitivity to thermal and tactile stimuli, causing substantial discomfort for affected children. The presence of these defects can significantly impact a child's quality of life, compromising both the esthetic and functional aspects of the involved dentition. The management of MIH often necessitates numerous dental interventions, which can exacerbate fear and anxiety in young patients and impose a considerable financial burden on families due to the requirement for extensive and ongoing restorative treatments [6, 8, 19].

Contemporary assessments of OHRQoL prioritize physical function, emotional experience, and an individual's social well-being, diverging from traditional medical or dental criteria [31]. Oral health factors are particularly important during adolescence, a critical period for self-perception development in young individuals [32]. In teenagers, oral health assessments can serve as a valuable guide for designing and implementing targeted oral health education strategies aimed at improving oral health status [33]. Parental perceptions also play a crucial role in this field and can be systematically evaluated using validated questionnaires [34].

This systematic review and meta-analysis summarized existing evidence on the impact of MIH on different domains of OHRQoL in teenagers and examined parental/caregivers perception.

Previous research [19] evaluating the effect of MIH on OHRQoL in 8–10-year-old children indicated a negative impact on the total score and all domains of the CPQ8-10 questionnaire, with the exception of social well-being. A similar systematic review conducted in 2022 [35], which did not include a meta-analysis, also concluded that MIH affects OHRQoL, particularly impacting the oral symptoms and functional limitation domains. Subsequent to the publication of this review, several new articles have contributed further evidence, thereby enabling a meta-analysis within the scope of the current review. The CPQ11-14 questionnaire was predominantly employed in the included studies [8, 13–15, 18, 23, 25] to evaluate the impact of MIH on OHRQoL. This questionnaire encompasses domains of oral symptoms, functional limitations, and emotional and social well-being [36]. For parental evaluations, the P-CPQ was utilized, assessing parents' perceptions of their children's oral health impact over the preceding 3 months. The P-CPQ comprises four domains: oral symptoms, functional limitation, emotional well-being, and social well-being [37, 38].

Although few studies have reported the negative impact of MIH on the OHRQoL of preadolescents [8, 13], some controversies still remain in this regard. Most of the studies that used the CPQ 11–14 reported no significant effect of MIH on OHRQoL [14, 15, 18, 23]. Nonetheless, some of them found MIH as affecting functional limitations [8, 13, 18, 25], oral symptoms [8, 13, 25], or emotional and social well-being [13, 25]. According to the C-OIDP questionnaire, MIH did not affect OHRQoL in children aged 6–16 years [24]. Conversely, other research, utilizing the C-OHIP questionnaire, indicated that suffering from MIH had a negative impact on OHRQoL, with this negative effect increasing in severity alongside MIH severity [26]. A clinical trial investigating the effect of minimally invasive esthetic treatments on children with MIH reported a pre-intervention C-OHIP score of 47 out of 76, with a significant improvement in this score and overall OHRQoL following the treatment [27].

Among the studies utilizing the CPQ11−14, only one study [14] included a matched control group based on the age and sex. Consequently, for the meta-analysis, we exclusively included the OHRQoL data from teenagers with MIH, without a direct comparison to a control group. Given that an ideal CPQ11−14 score is zero, all reported scores were compared against this baseline.

This meta-analysis revealed that MIH can negatively impact OHRQoL in teenagers, primarily through its effects on the oral symptoms and functional limitations domains. This suggests that teenagers with MIH experienced symptoms such as toothache, sensitivity while eating cold food, food sticking, and bad breath at least once in the preceding month. They also reported functional limitations, including sleep disturbances due to dental issues and difficulties with biting, chewing, and clear pronunciation. However, teenagers with MIH did not report a significant decline in the social and emotional well-being. Also, they did not express discomfort from social well-being factors such as laughing or talking. This might imply that, despite experiencing oral symptoms and functional limitations, teenagers may not feel uncomfortable or ashamed of their dental condition. Meta-analysis results indicated a negative effect of MIH on the total score of OHRQoL in teenagers, underscoring the importance of early diagnosis and intervention to improve their perception of their oral health status.

While parental reports can complement the child statements regarding quality of life in various studies, some researchers express concern that parents may not accurately reflect the child's perception [34, 39]. Nevertheless, parental reporting provides essential information and should not be disregarded [17]. In the context of the current study, despite children experiencing a significant negative impact of MIH on their OHRQoL, parents/caregivers did not appear to fully perceive these problems. This potential parental misconception could lead to a lack of appropriate planning for the prevention or treatment of MIH.

A previous study indicated no significant effect of MIH on OHRQoL from the parents' perspective [15]. Arrow et al. [17] reported that parents perceived MIH as having no significant impact on the OHRQoL of their affected 7-year-old children. Conversely, Dias et al. [18] demonstrated that in 6–11 years children with severe MIH, a greater negative impact was perceived within the functional limitation and emotional well-being domains. Dantes-neta et al. [8] similarly found that according to the parent/caregivers' perceptions using P-CPQ, 11–14 years children with severe MIH experienced greater functional limitation compared to those without MIH.

The findings of the present meta-analysis indicated that the effect of MIH on all four domains and the total score of the P-CPQ was not statistically significant. This outcome may be attributable to the limited number of studies examining parental perceptions or variations in the age groups of the children included.

A limitation of this study was the inconsistency in the quality of life assessment tools used across different studies, which precluded the inclusion of a larger number of studies in the meta-analysis. Consequently, further research employing standardized quality of life assessment tools, such as the COHIP or ECOHIS, is imperative to facilitate more reliable comparisons and robust meta-analyses. Future investigations should focus on specific age cohorts (e.g., 6–8 and 12–15 years) to meticulously track MIH's impact across distinct developmental stages. Longitudinal investigations are crucial for elucidating etiological factors, understanding disease progression, and evaluating the long-term efficacy of various treatment modalities, including their psychological and financial implications. A multidisciplinary approach, integrating dental, psychological, and economic perspectives, is also recommended for a comprehensive understanding and management of MIH.

Furthermore, we advocate for future studies to place greater emphasis on parental perceptions of children affected by MIH, particularly younger children. Such data, when combined with children's reports, can significantly aid dentists and health policymakers in determining optimal support and treatment strategies for these children.

## 5. Conclusion

Based on the present study, the presence of MIH decreased the OHRQoL in teenagers. This negative impact was significant in the total score of CPQ 11–14 and oral symptom and functional limitation domains. However, parents/caregivers did not perceive this negative impact on their children's OHRQoL.

## Figures and Tables

**Table 1:** Evidence table of studies included in the systematic review.

Authors/country	Study design	Sample size (*N*)	Age (years)	Gender (M: male, F: female)	With MIH	MIH diagnose	MIH subgroups based on severity (*N*)	Severe MIH (*N*)	Mild MIH (*N*)	OHRQoL measurement tool	Conclusion
Arrow [17]/ Australia	Cross-sectional	550	7	M = 248, F = 302	399	DDE index	No enamel defects; diffuse/demarcated defects; diffuse/ demarcated defects with hypoplasia(*n* = 3)	54 /demarcated	335	P-CPQ	No impact of MIH on OHRQoL is shown
Arrow [23]/ Australia	Cross-sectional	88	14–16	NR	NR	DDE index	Diffuse/diffuse and hypoplastic/demarcated/demarc and hypoplastic/pits (*n* = 5)	18	NR	CPQ11-14	No impact of MIH on OHRQoL is shown
Dantas-Neta et al. [8]/ Brazil	Cross-sectional	594	11–14	NR	109	EAPD 2003	Mild/moderate/severe (*n* = 3)	25	84	P-CPQ CPQ11-14	Severe MIH was significantly associated with a greater negative impact of the “functional limitation domain” according to parents'/caregivers' perceptions. According to the children, severe MIH was significantly associated with a greater negative impact of the “oral symptom domain” and “functional limitation domain”
Dias et al. [18]/ Brazil	Cross-sectional	42 (6–7 years) 211 (8–11 years)	6–11	M = 125, F = 128	253	EAPD 2003	Mild/severe (*n* = 2)	15	238	CPQ8-10 CPQ11-14 P-CPQ	MIH has a certain but not significant impact on the domains: “Oral symptoms,” “Functional limitations” and also “Emotional wellbeing” (the parents). MIH had no significant impact on OHRQoL according to children's perceptions
Folayan et al. [24]/ Nigeria	Cross-sectional	428 (6–9 years) 425 (10–16 years)	6–16	M = 438, F = 415	20 5	EAPD 2003	MIH presence (*n* = 1)	20 5	NR	Child OIDP	No impact of MIH on OHRQoL is shown
Portella et al. [14]/ Brazil	Case–control	93	6–13	M = 62, F = 31	31	EAPD 2003	Mild/severe (*n* = 2)	NR	NR	CPQ8-10 CPQ11-14	No impact of MIH on OHRQoL was observed. No significant difference was found between the overall mean CPQ8−10 score in relation to MIH severity
Hasmun et al. [27]/ United Kingdom	Clinical trial	103	7–16	M = 62, F = 41	103	EAPD 2003	MIH presence (*n* = 1)	NR	NR	C-OHIP-SF19	Uncomplicated, minimally invasive dental procedures might improve children's wellbeing and OHRQoL by reducing the appearance of enamel opacities in MIH (only the before procedure reported OHRQoL of MIH-affected children was used)
Shojaeepour et al. [13]/ Iran	Cross-sectional	2507	8–12	M = 1795, F = 712	129	EAPD 2003	Severe/mild (*n* = 2)	82	47	CPQ8-10 CPQ11-14	MIH had a significant negative impact on OHRQoL (*p* <0.001)
Freitas Fernandes et al. [15]/ Brazil	Cross-sectional	463	11–14	M = 170, F = 293	50	Ghanim 2017	Severe/mild (*n* = 2)	28	22	B-P-CPQ CPQ11-14	According to students and their parents/caregivers' perception, incisor molar hypomineralization did not influence OHRQoL of the studied sample
Elhennawy et al. [26]/ Germany	Cross-sectional	317	7–14	M = 161, F = 156	217	EAPD 2003	Mild/severe (*n* = 2)	157	60	COHIP-G19	MIH affects OHRQoL significantly in all domains. This negative effect is greater for severe MIH (*p* <0.001)
Tugcu et al. [25]/ Turkey	Cross-sectional	40	11–14	M = 16, F = 24	40	Ghanim	Severe MIH (*N* = 1)	40	0	CPQ11-14	Children with severe molar-incisor hypomineralization who received restorative therapy with glass hybrid restorative system after selective caries removal reported improvements in their OHRQoL (only the before procedure reported OHRQoL of MIH-affected children was used)

Abbreviation: NR, not reported.

## Data Availability

The data that support the findings of this study are available on request from the corresponding author.
